# Boosting the Lithium-Ion Conductivity in Li_7_TaP_4_ by Aliovalent Li versus Ta Substitution by Three Orders of Magnitude

**DOI:** 10.1021/acs.inorgchem.5c02167

**Published:** 2025-08-11

**Authors:** Samuel Merk, Simon Kollmannsberger, Sabine Zeitz, Volodymyr Baran, Anatoliy Senyshyn, Thomas F. Fässler

**Affiliations:** † Technical University of Munich (TUM), TUM School of Natural Sciences, Department of Chemistry, Chair of Inorganic Chemistry with Focus on New Materials, Lichtenbergstraße 4, Garching D-85748 Germany; ‡ TUMint.Energy Research GmbH, Lichtenbergstraße 4, Garching D-85748, Germany; § 28332 Deutsches Elektronen Synchrotron (DESY), Notkestr. 85, Hamburg 22607, Germany; ∥ Research Neutron Source Heinz Meier-Leibnitz (FRM II), Technische Universität München, Lichtenbergstraße 4, Garching bei München 85747, Germany

## Abstract

Lithium-ion conductors are one of the key features of all-solid-state lithium-ion batteries. To modify their properties and enable their implementation in high-performance devices, an understanding of the relationship between the crystal structure and the transport properties of the mobile species is important. Lithium phosphidotetrelates and -trielates are classes of lithium-ion conductors reaching ionic conductivities of up to 4.5 × 10^–3^ cm^–1^ at room temperature for ω-Li_9_GaP_4_. Here, we present the new lithium phosphidotantalate Li_7_TaP_4_, and the aliovalent substitution of Ta by Li atoms, which leads to a partial filling of octahedral voids in the structure of Li_7_TaP_4_. As a result, the lithium-ion conductivity of Li_7_TaP_4_ (1.3 × 10^–7^ S cm^–1^) increases by 3 orders of magnitude to 3.7 × 10^–4^ S cm^–1^ in Li_9.5_Ta_0.5_P_4_. Li_7_TaP_4_ and Li_9.5_Ta_0.5_P_4_ crystallizing in the cubic space groups *Pa*3̅ and *Fm*3̅*m*, respectively, show a close structural relationship. The structure-property relationship is highlighted and compared with the isotypic tetrel element analogues.

## Graphical abstract

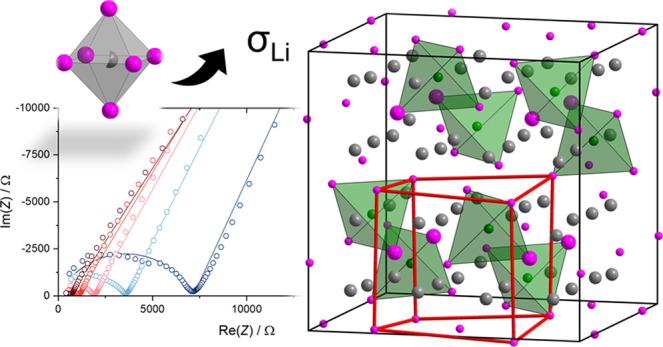

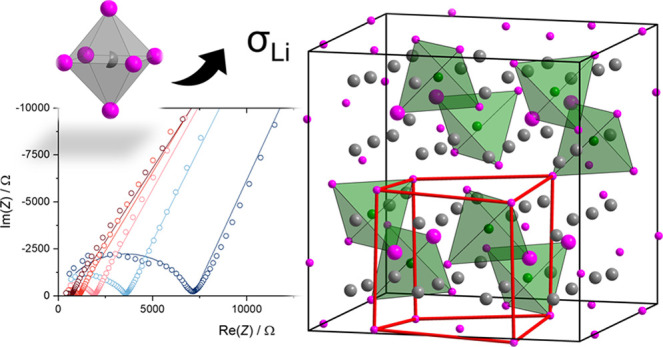

## Introduction

All-solid-state batteries (ASSBs) have been investigated intensively as an alternative for conventional liquid batteries, enhancing key features like safety, energy/power density, and mechanical stability.^1^ The solid electrolyte is one of the key components for this technology.^2^ In addition to electrochemical stability, efficient ion conduction is a key parameter that ensures fast charging and a high current density in solid-state batteries. In this context, it is crucial to explore how materials can be engineered to enhance their ionic conductivity. Over the previous years, we investigated phosphide-based materials that have been proven to resemble a large family of ionic conductors leading to the discovery of a variety of lithium phosphidotetrelates and -trielates reaching ionic conductivities of up to 4.5 × 10^–3^ S cm^–1^ at room temperature for ω-Li_9_GaP_4_.^3–11^ In a broader view, the huge potential of pnictogen-based Li-ion conductors has been recently shown for Li_3–3x_Sc_*x*_Sb reaching an ionic conductivity of 4.2 × 10^–2^ S cm^–1^.^12^ The general concept of the phosphide-based materials is the presence of highly negatively charged [*Tt*/*Tr*P_4_]^8‑/9–^ building units (*Tt* = tetrel or Group-14 element, *Tr* = triel or Group-13 element) that are able to accommodate more lithium compared to other electrolyte classes like sulfides, oxides, or halides.

The crystal structures of Li_8_ *Tt*P_4_ are closely related to the Li_3_Bi type, which features cubic close packing (*ccp*) of phosphorus atoms. This arrangement generates eight tetrahedral and four octahedral voids per formula unit comprising four phosphorus atoms. Of these 12 voids in sum, only nine are occupied. A covalently bonded tetrel element occupies one tetrahedral void, and eight lithium atoms are distributed over the remaining 11 voids, leaving several vacancies to facilitate ionic motion within the system. Since the tetrahedral and octahedral voids share faces, the diffusion pathways can occur through triangles that merge neighboring voids and are wider compared to scenarios with only edge-sharing polyhedra. Further research revealed the discovery of Li_14_ *Tt*P_6_ (*Tt* = Si, Ge, Sn),^8,9^ representing the highest lithium content in this class of materials. The formal addition of two Li_3_P units to Li_8_ *Tt*P_4_ results in complete disorder of lithium and tetrel atoms in the tetrahedral positions. Additionally, the aliovalent substitution of tetrel elements with triel elements was explored. Increasing the number of charge carriers in Li_9_ *Tr*P_4_ (*Tr* = Al, Ga, In) results in crystal structures very similar to those of phosphidotetrelates and achieves even higher ionic conductivities, as previously mentioned.

Moving from main group elements to transition metals enables a variety of different compounds. Numerous lithium transition metal phosphides with different structural motifs are reported.^13^ Lithium-poor transition metal (*T*) phosphides comprise different coordination spheres of the transition metal atoms, such as CoP_5_ quadratic pyramids in LiCo_6_P_4_ and *T*P_4_ tetrahedra as well as *T*P_6_ octahedra in Li_2_ *T*_12_P_7_ (*T* = Co, Ni).^14,15^ At higher lithium content exclusively edge-sharing tetrahedral *T*P_4_ units are observed, which form layers and three-dimensional polyanionic networks.^16–19^ Furthermore, copper phosphides tend to form planar heterographite networks and double layers, respectively.^17,20^

Lithium-rich transition-metal phosphides comprise isolated tetrahedral *T*P_4_ units, and their structures can be derived from a cubic close packing of the phosphorus atoms. While the transition metal solely occupies the tetrahedral voids, lithium occurs in both tetrahedral and octahedral voids, resulting in antifluorite and ordered Fe_3_Al-type structures, respectively.^16,21–26^ We refer here to the structures of the ionic representatives Li_2_O and Li_3_Bi, respectively, as boundary representatives in which either all tetrahedral voids or both tetrahedral and octahedral voids are fully occupied by Li ions. In contrast to their analogous Group-5 lithium metal nitrides, which form superstructures of the Li_2_O type,^27^ the known representatives of Group-5 lithium metal phosphides commonly crystallize in the Li_2_O structure with a statistical occupation of all tetrahedral voids with Li and *T* atoms and all octahedral voids remain empty as observed in Li_7_VP_4_, Li_7_NbP_4_ and Li_7.5_Ta_0.5_P_4_. On the other hand, octahedral voids are partially filled in the case of Li_9.8_V_1.2_P_4_ which thus is assigned to a defect variant of the Li_3_Bi structure type.^21–23,28^ Generally, octahedral vacancies are gradually filled when lithium in tetrahedral voids is replaced by transition metals with an oxidation state higher than +1. Independent of the structure type, the stoichiometry of lithium-containing phases—based on four formula units of Li_3_P assuming the Li_3_Bi type—can be expressed by the general formula ${\mathrm{Li}}_{12-n\cdot x}^{+}T_{x}^{n+}{□}_{x\cdot \left(n-1\right)}P_{4}^{3-}$ , where *T* ^*n*^+ denotes a transition metal with oxidation state *n* and □ a vacancy. For *x* = 0, the pristine formula (Li_3_P)_4_ results. Substituting *n* Li^+^ ions with one *T* ^*n+*^ ion results in the formation of (*n* – 1) vacancies, depending on the valency *n* of the transition metal. Li_7_VP_4_, Li_7_NbP_4_ and Li_7.5_Ta_0.5_P_4_ that crystallize in the Li_2_O type can therefore be expressed as ${\left({\mathrm{Li}}_{7}T_{1}\right)}_{tet}{\left({□}_{4}\right)}_{oct}P_{4}$ and ${\left({\mathrm{Li}}_{7.5}T_{0.5}\right)}_{tet}{\left({□}_{4}\right)}_{oct}P_{4}$ , respectively, with the subscript tet and oct indicating the species within the tetrahedral and octahedral voids. In Li_9.8_V_1.2_P_4_, the overall number of transition metal and lithium atoms exceeds the number of tetrahedral voids. Consequently, the occupation of the octahedral voids results, which however was not further investigated by Juza et al. Full occupation of the tetrahedral voids and filling of the octahedral voids with remaining lithium would result in ${\left({\mathrm{Li}}_{6.8}T_{1.2}\right)}_{tet}{\left({\mathrm{Li}}_{3}\right)}_{oct}{\left({□}_{1}\right)}_{oct}P_{4}$ denoting that 6.8 Li and 1.2 *T* atoms are located in tetrahedral voids, which are then filled by 100%, and three Li atoms are situated in octahedral voids, whereas one-quarter of the existing octahedral voids remains unoccupied.^29^

In the following, we report on the investigation of the system Li_12–5*x*_Ta_*x*_P_4_ (0.25 ≤ *x* ≤ 1) including the compounds Li_9.5_Ta_0.5_P_4_ and Li_7_TaP_4_ for *x* = 0.5 and 1.0, respectively. A two-step synthesis including mechanochemical ball milling and subsequent annealing enables the phase-pure synthesis of the two new ternary compounds Li_9.5_Ta_0.5_P_4_ and Li_7_TaP_4_. Detailed characterization was done by synchrotron X-ray powder diffraction, complemented by the Rietveld method, differential scanning calorimetry (DSC), solid-state magic angle spinning (MAS) NMR spectroscopy, and Raman spectroscopy. Additionally, the Li^+^ mobility and its activation energy were determined by electrochemical impedance spectroscopy (EIS).

## Experimental Section

All steps of synthesis and sample preparation were performed inside an argon-filled glovebox (MBraun, *p*(H_2_O), *p*(O_2_) < 1.2 ppm) or in sealed containers under an Ar atmosphere. Before use, lithium (Li, rods, Rockwood Lithium, > 99%) was cleaned from surface impurities. Tantalum (Ta, powder, Thermo Scientific, 99.98%) and red phosphorus (P, powder, Sigma-Aldrich, 97%) were used without further purification. All obtained compounds are sensitive to oxygen and moisture, with the latter showing vigorous reactions that result in flammable and toxic gases.

### Synthesis of Li_7_TaP_4_ and Li_9.5_Ta_0.5_P_4_

The series ${\mathrm{Li}}_{12-5x}{\mathrm{Ta}}_{x}P_{4}$ (*x* = 0.25, 0.5, 0.75, and 1) was prepared in a two-step synthesis from the elements via ball milling and subsequent annealing. Batches of the “reactive mixture” with *m* = 3.0 g containing lithium, tantalum, and red phosphorus were prepared by mechanochemical milling (Retsch PM 100 planetary mill, 18 h, 350 rpm, intervals of 10 min with direction reversal and subsequent 5 min resting) using a WC milling set (50 mL jar with 3 balls with a diameter of 15 mm each). The respective weigh-ins are listed in Table 1.

**Table 1:** Weigh-Ins of the Reactive Mixtures of Li_12–5*x*_Ta_*x*_P_4_ (*x* = 0.25, 0.5, 0.75, and 1)

Li12_–_5_*x*_Ta_x_P_4_	Lithium *m*, *n*, *equiv*	Tantalum *m*, *n*, *equiv*	Phosphorus *m*, *n*, *equiv*
*x* = 0.25	918.3 mg, 132.3 mmol, 43 equiv	556.8 mg, 3.08 mmol, 1 equiv	1524.9 mg, 49.23 mmol, 16 equiv
*x* = 0.5	705.6 mg, 101.68 mmol, 19 equiv	968.3 mg, 5.35 mmol, 1 equiv	1326.0 mg, 42.81 mmol, 8 equiv
*x* = 0.75	542.1 mg, 78.11 mmol, 11 equiv	1284.9 mg, 7.1 mmol, 1 equiv	1173.0 mg, 37.87 mmol, 5.33 equiv
*x* = 1.0	412.4 mg, 59.42 mmol, 7 equiv	1536 mg, 8.49 mmol, 1 equiv	1051.7 mg, 33.95 mmol, 4 equiv

The obtained black “reactive mixtures” were sealed into tantalum crucibles in batches of 500 mg using an arc furnace (Edmund Bühler MAM1). The sealed ampules were enclosed in evacuated silica reaction containers. The containers were heated in a tube furnace (HTM Reetz Loba) with 4 °C min^–1^ up to 650 °C for 24 h and to 550 °C for 46 h for *x* = 0.5 and 1, respectively. To obtain phase-pure samples of Li_9.5_Ta_0.5_P_4_ (*x* = 0.5) and Li_7_TaP_4_, (*x* = 1.0), the sealed ampules were quenched to room temperature in water. After grinding, black (Li_9.5_Ta_0.5_P_4_) and brown powders (Li_7_TaP_4_) were obtained.

To investigate the possibility of a phase width, Li_12–5*x*_Ta_*x*_P_4_ was further explored for *x* = 0.25 and 0.75. Similar to the synthesis route for Li_7_TaP_4_ and Li_9.5_Ta_0.5_P_4_, stoichiometric mixtures of the elements were ball-milled in a first step and heat-treated in the second step at three different temperatures (550, 700, and 900 °C). The reactive mixtures after ball milling contained a mixture of metallic Ta and Li_3_P (Figure S1). Annealing the mixture “Li_10.75_Ta_0.25_P_4_” leads to the formation of Li_3_P and Li_9.5_Ta_0.5_P_4_ at all three temperatures with some residual Ta within the sample (Figure S2). At 900 °C, a couple of unknown reflections appear. In contrast to that, during annealing, the mixture “Li_8.25_Ta_0.75_P_4_” at 550 °C, Li_7_TaP_4_, Li_9.5_Ta_0.5_P_4_, and TaP form (Figure S3). At higher temperatures, Li_7_TaP_4_ decomposes to Li_9.5_Ta_0.5_P_4_ and TaP. In addition, unknown reflections can be observed. Indexing of the emerging reflections in the diffractograms of Li_7_TaP_4_ and Li_9.5_Ta_0.5_P_4_ suggests that Li_7_TaP_4_ is a stoichiometric compound. The indexed lattice parameters do not differ significantly according to the 3σ rule (Figure S8). For Li_9.5_Ta_0.5_P_4_ a small decrease of the lattice parameter with a higher Ta content according to Li_12–*x*_Ta_*x*_P_4_ for *x* = 0.25, 0.50, and 0.75 is observed. This hints for a narrow phase width. However, due to the presence of side products and resulting uncertainty in the actual *x* value, the exact phase width was not further investigated.

### Powder X-ray Diffraction

For powder X-ray diffraction (PXRD) measurements, the samples were ground in an agate mortar and sealed inside 0.3 mm glass capillaries. PXRD measurements were performed at room temperature on a STOE Stadi P diffractometer (Ge(111) monochromator, Cu *Kα*_1_ radiation, λ = 1.540598 Å) with a Dectris MYTHEN 1K detector in a Debye–Scherrer geometry. The raw powder data were processed with the software package WinXPOW.^30^

### Synchrotron X-ray Data and Rietveld Refinement

The samples for the powder X-ray synchrotron diffraction measurements were filled with capillaries with a diameter of 0.5 mm. PXRD measurements were performed on the P02.1 beamline^31^ at the PETRA III Synchrotron (DESY, Hamburg, Germany). The data were collected using a Varex XRD 4343CT detector with a 150 μm × 150 μm pixel size. The distance between the detector and the sample was fixed at 1.503 m. The energy of the synchrotron radiation was set at 60 keV (λ = 0.20707 Å). The reference NIST SRM 660c LaB_6_ is used as a standard powder for the calibration of the diffraction data. Data calibration and integration were done using the pyFAI software.^32^

Rietveld refinements of Li_7_TaP_4_ and Li_9.5_Ta_0.5_P_4_ were executed using the full-profile Rietveld method within the FullProf program package.^33^ For Li_7_TaP_4_ and Li_9.5_Ta_0.5_P_4_ the structure models of α-Li_8_GeP_4_ and Li_14_SiP_6_ were used as an input. The Thompson–Cox–Hastings profile function was used to model the peak profile shape. Background contribution was determined using a linear interpolation between selected data points in nonoverlapping regions. The scale factor, profile shape parameters, resolution (Caglioti) parameters, and lattice parameters as well as fractional coordinates of atoms were refined freely. For Li_7_TaP_4_, the site occupancies of all atoms were set to one. For Li_9.5_Ta_0.5_P_4_, the tetrahedral and octahedral void occupations were refined freely.

### Differential Scanning Calorimetry (DSC)

For thermal analysis, samples were sealed in a niobium ampule and measured on a DSC machine (Netzsch, DSC 404 Pegasus) under a constant gas flow of 75 mL min^–1^. The reactive mixtures of Li_7_TaP_4_ and Li_9.5_Ta_0.5_P_4_ were heated to 600 and 800 °C, respectively, and cooled to 150 °C twice at a rate of 5 °C min^–1^. To study the thermal stability of the pure compounds, Li_7_TaP_4_ and Li_9.5_Ta_0.5_P_4_ were heated to 1000 °C and cooled to 150 °C twice at a rate of 5 °C min^–1^. Data were processed using the PROTEUS Thermal Analysis software.^34^

### Raman Spectroscopy

Measurements were carried out at room temperature by using samples sealed in glass capillaries with a diameter of 0.3 mm. To ensure reproducibility, different spots of the capillary were measured. The measurements were performed using an *inVia* Reflex Raman (Renishaw) system equipped with a CCD Master:Renishaw 266n10 detector (Renishaw) coupled to a Leica DM2700 M microscope (Leica) with 50× magnification and both a 532 and a 785 nm laser. The samples were measured for 1 s, repeated 100 times (total measuring time: 100 s). For operating the device and data handling, the software WiRE 5.3 (Renishaw) was used.^35^ The installed Rayleigh filter cuts off signals below 110 cm^–1^.

### DFT Analysis

The computational analysis of Li_7_TaP_4_ was performed using the CRYSTAL17 program package and hybrid density functional methods.^36,37^ A hybrid exchange-correlation functional after Perdew, Burke, and Ernzerhof (DFT-PBE0) was used.^38^ Localized, Gaussian-Type triple ζ-valence + polarization level basis sets were used for Ta and P and split valence + polarization level basis sets were used for Li. The basis sets were derived from the molecular Karlsruhe basis sets.^39–41^ For the evaluation of the Coulomb and exchange integrals (TOLINTEG), tight tolerance factors of 8, 8, 8, 8, and 16 were used for all calculations. The reciprocal space of the structure was sampled with a 3 × 3 × 3 Monkhorst–Pack-type k-point grid. The starting geometry was taken from the experimental data. Both the lattice parameters and atomic positions were fully optimized within the constraints imposed by space symmetry. Furthermore, the optimized structure was confirmed to be true local minimum by means of harmonic frequency calculation at the Γ-point. The electronic band structure and density of states (DOS) were calculated, as well as the crystal orbital Hamilton population for all heteroatomic interactions. The Brillouin Zone path of Γ—X—M—Γ—R—X|R—M—X_1_ was provided by the web service SeeK-path.^42^ Using the results of the frequency calculation, a theoretical Raman spectrum was calculated by utilizing an analytical CPHF/CPKS scheme (coupled perturbed Hartree–Fock/Kohn–Sham). The full width at half-maximum (fwhm) was set to 8 cm^–1^, the pseudo-Voigt broadening to 50:50 Gaussian:Laurenzian and the laser wavelength to 785 nm. To assign signals in the spectrum to vibrations of the lattice, the platform CRYSPLOT^43^ was used for visualizing the theoretical vibration modes. Since the calculations are based on the structure at 0 K, there are discrepancies in intensities compared to measurements at room temperature. Additionally, a correction factor of 0.96 has been applied to adjust for overestimating wavenumbers at high frequencies.^44^

### MAS-NMR Spectroscopy

Magic angle spinning nuclear magnetic resonance (MAS-NMR) spectra were recorded on a Bruker Avance 300 NMR instrument operating at 7 T. A 4 mm ZrO_2_ rotor was filled inside a glovebox and subsequently rotated and cooled in a nitrogen stream during the measurement. The resonance frequencies for^6^Li and ^31^P are 44.2 and 121.5 MHz, respectively. The rotation frequency was set to 15 kHz. The ^6^Li spectra were referred to lithium chloride with a chemical shift of −1.15 ppm referenced to 1 M LiCl in H_2_O (δ = 0 ppm). The ^31^P spectra were referred to (NH_4_)­H_2_PO_4_(s) (ammonium dihydrogen phosphate) with a chemical shift of 1.11 ppm with respect to concentrated H_3_PO_4_(aq) (phosphoric acid). All spectra were recorded by using single-pulse excitation. Raw data were edited and evaluated with MestreNova.^45^

### Impedance Spectroscopy and DC Conductivity Measurements

The ionic conductivities of Li_7_TaP_4_ and Li_9.5_Ta_0.5_P_4_ were determined by electrochemical impedance spectroscopy (EIS) in a commercial cell with tungsten carbide electrodes (RHD Instruments, CompreCell). Measurements were conducted on three independent samples for each compound to ensure reproducibility. Powdered samples (200 mg) were compressed in a hydraulic press with a pressure of 400 MPa to at least 70% of the crystal density. During electrochemical measurements, a constant pressure of 150 MPa was applied on the cell by the compression of springs. Impedance spectra were recorded on a Bio-Logic potentiostat (VMP300) in a frequency range from 7 MHz to 100 mHz at a potentiostatic excitation of ±50 mV. Data were treated using the software RelaxIS.^46^ The measurements were performed in a climate cabinet (ESPEC, model LU-114). For the determination of the activation energy of lithium-ion conduction, the cell temperature was set to 10, 25, 40, 50, and 70 °C. Prior to EIS measurements, the cell rested 150 min to allow for thermal equilibration. The electronic conductivity was determined with the same setup using potentiostatic polarization applying voltages of 50, 100, and 150 mV for 8 h each.

## Results and Discussion

### Synthesis and Structure Determination of Li_7_TaP_4_ and Li_9.5_Ta_0.5_P_4_

Li_7_TaP_4_ and Li_9.5_Ta_0.5_P_4_ were synthesized by ball milling of the elements and subsequent annealing at 550 and 650 °C, respectively, followed by rapid cooling to room temperature in water. The powder XRD images of the reactive mixtures after ball milling are shown in Figure S1. The crystal structures of Li_7_TaP_4_ and Li_9.5_Ta_0.5_P_4_ were solved and refined by means of Rietveld refinement (Figure 1) using synchrotron X-ray diffraction data for both compounds. Detailed crystallographic data and the outcomes of the Rietveld refinements are provided in Table 2 and Tables S1–S4.

**Figure 1:**
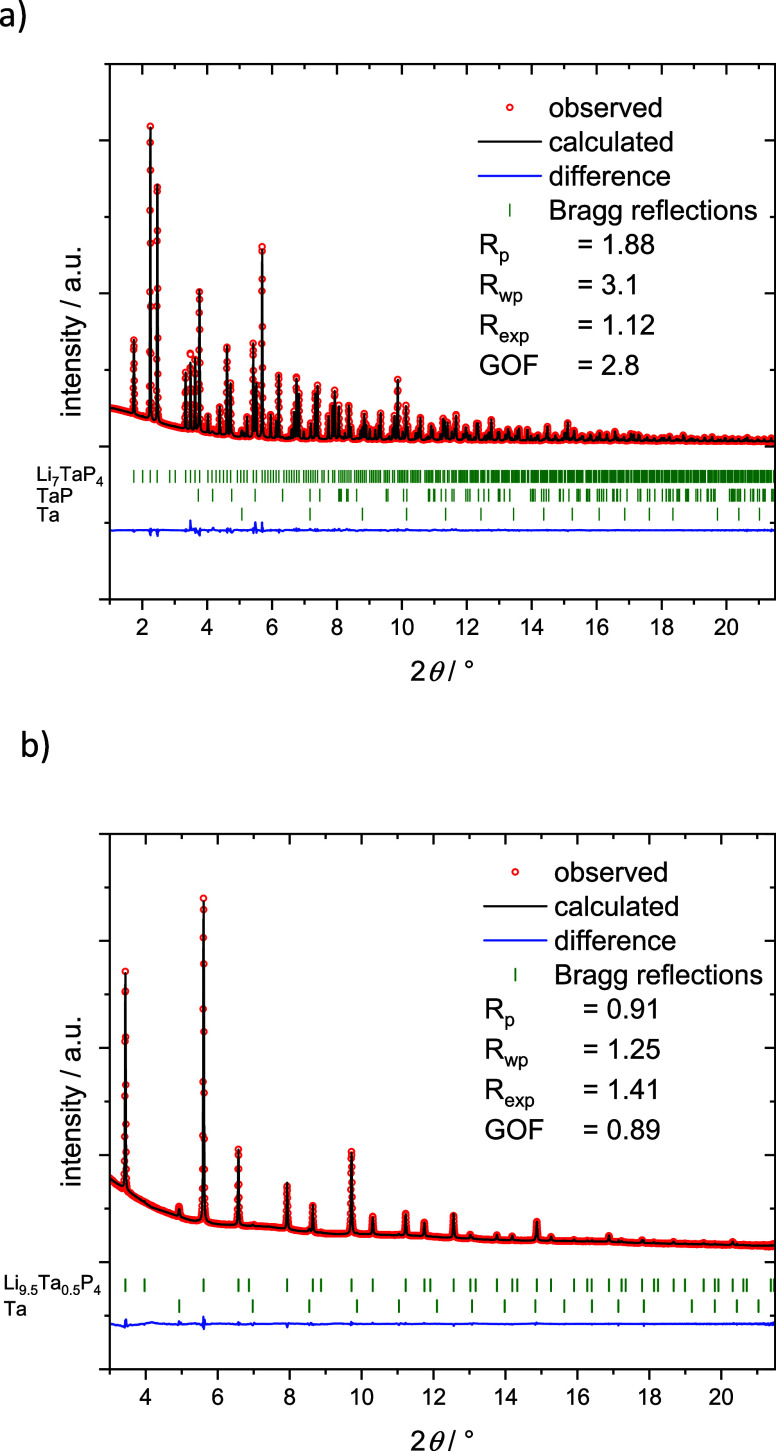
Rietveld refinement of the powder X-ray pattern of a) Li_7_TaP_4_ and b) Li_9.5_Ta_0.5_P_4_. The red circles indicate observed intensities, the black line shows the calculated intensities, and the blue line shows the difference. Bragg positions are depicted as green dashes. The respective ratios are Li_7_TaP_4_: TaP: Ta 96.5(2) wt %: 3.0(1) wt %: 0.5(1) wt % as well as Li_9.5_Ta_0.5_P_4_: Ta 99.8(9) wt %: 0.2(1) wt %.

**Table 2:** Crystallographic Data of Li_7_TaP_4_ and Li_9.5_Ta_0.5_P_4_ Obtained by Rietveld Analysis of the Synchrotron Powder Diffraction Data

Empirical Formula	Li_7_TaP_4_	Li_9.79(9)_Ta_0.451(6)_P_4_
*T*/K	293	293
Formula weight/g mol^–1^	353.4	273.7
Space group (no)	*Pa*3̅ (205)	*Fm*3*m* (225)
Unit cell parameters/Å	*a* = 11.80105(1)	*a* = 5.992740(8)
*Z*	8	1
*V* /Å^3^	1643.469(3)	215.2169(5)
ρ_calc._/g cm^–3^	2.85665	2.11188
2θ range/deg	1–21.5	3–21.5
*R*_p_	1.88	0.91
*R*_wp_	3.1	1.25
*R*_exp_	1.12	1.41
Χ^2^	8.23	1.08
*R*_Bragg_	2.5	7.25
*R*_f_	2.3	8.17
*Depository no.*	CSD-2448967	CSD-2448969

Li_7_TaP_4_ crystallizes in the cubic space group *Pa*3̅ (no. 205) with a lattice parameter of *a* = 11.80105(1) Å at room temperature and comprises isolated TaP_4_ tetrahedra, which are separated by seven Li atoms per formula unit. Assuming positively charged Li ions, [TaP_4_]^7–^ tetrahedra result (Figure 2). Li_7_TaP_4_ is a defect variant of Li_8_SiP_4_ and is isotypic to Li_7_TaN_4_, which appears as a 2 × 2 × 2 superstructure of the closely related Li_2_O type and features the same group-subgroup relationships as reported for the lithium phosphidotetrelates.^3,47^ Moreover, Li_7_TaP_4_ features six fully occupied and crystallographically independent atom positions (Li1, Li2, Li3, Ta, P1, and P2). The P atoms arrange in a slightly distorted *ccp* configuration, with all tetrahedral voids fully occupied by Ta (8*c*) and Li (Li1 8*c*, Li2 24*d*, and Li3 24*d*) in an ordered manner, maintaining a ratio of 1:7. In contrast to the isotypic lithium phosphidotetrelates, a second set of 24*d* positions as well as the 4*a* and 4*b* sites, all corresponding to octahedral voids, remain entirely vacant. Similar to the structural configurations observed in Li_8_SiP_4_, the arrangement of Ta atoms within the unit cell aligns with the shape of a rhombohedron. Owing to the space group symmetry and the distorted *ccp* arrangement of the P atoms, each Ta atom adopts a distorted tetrahedral coordination, surrounded by one P1 and three P2 atoms, resulting in slightly varied Ta–P distances of 2.392(6) Å for Ta–P1 and 2.385(3) Å for Ta–P2, alongside P–Ta–P bond angles deviating from the ideal tetrahedral bond angle of 109.5° (measured as 109.3(3)° and 109.7(2)°). These Ta–P distances are similar to previously observed distances in compounds with isolated TaP_4_-units ranging from 2.38 to 2.44 Å.^48–51^ Analysis of interatomic distances reveals comparable distances between the central atom and the surrounding P atoms (ranging from 2.5 to 2.75 Å) for Li1–Li3 atoms in tetrahedral voids. Examination of the resulting coordination polyhedra, as depicted in Figure S9, suggests the presence of slightly distorted (Ta/Li)­P4 tetrahedra.

**Figure 2:**
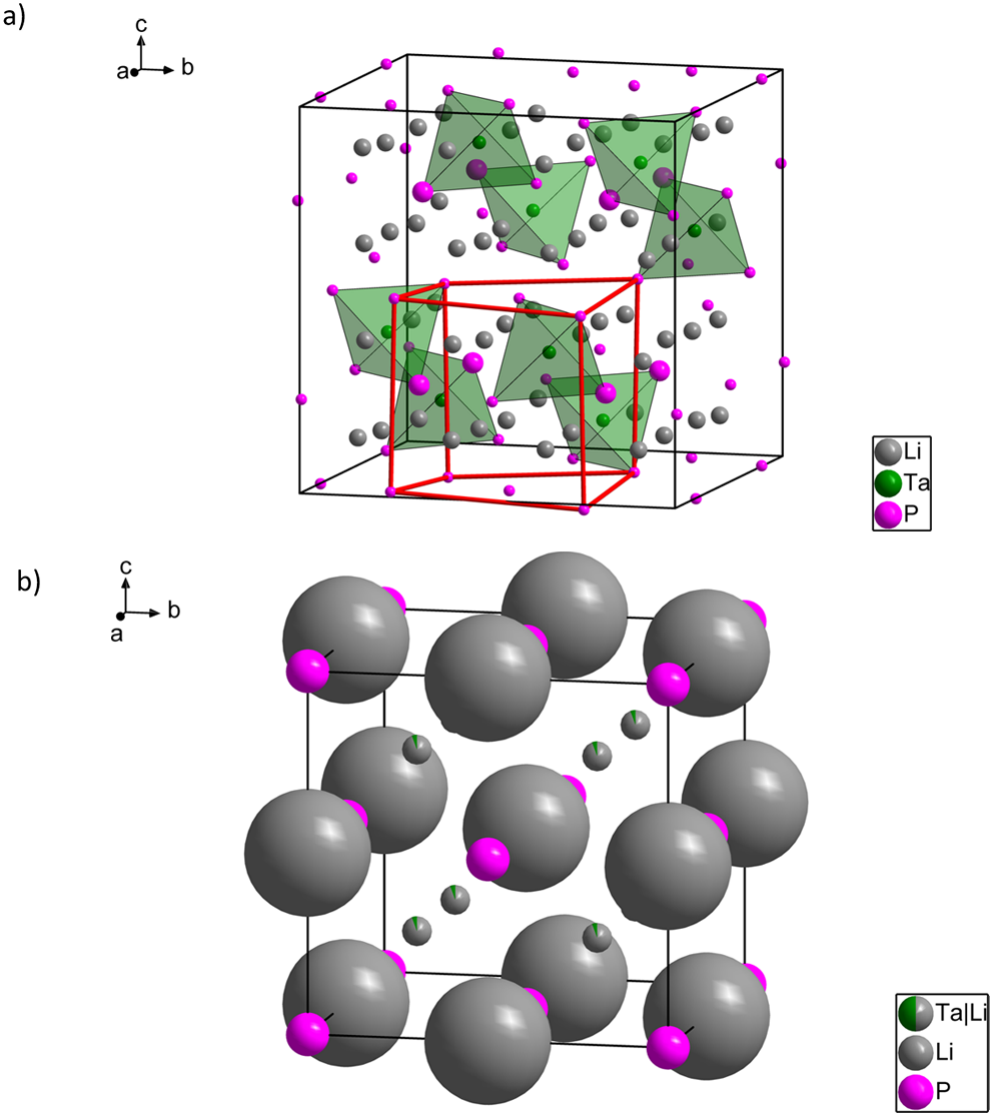
Unit cells of a) Li_7_TaP_4_ and b) Li_9.5_Ta_0.5_P_4_. Notice that the dimensions of the unit cells are not drawn to scale. The distorted face-centered cubic arrangement of the P atoms in Li_7_TaP_4_ is emphasized in red. In Li_9.5_Ta_0.5_P_4_, the octahedral voids are occupied by 57(2)% with Li. Li, Ta, and P are depicted in gray, green, and pink, respectively. The displacement ellipsoids are set at 90%.

The Rietveld analysis of the synchrotron X-ray diffraction of Li_9.5_Ta_0.5_P_4_ reveals a cubic structure crystallizing in the space group *Fm*3̅*m* (no. 225) with a lattice parameter of *a* = 5.994796(8) Å at room temperature, thus according to the Li_3_Bi type formed by three crystallographic atom positions (P, Li1/Ta, Li2). The structure comprises a *ccp* of P atoms, and all tetrahedral voids are statistically filled with Li1 and Ta atoms. The remaining Li atoms Li2 fill 57(2)% of the octahedral voids. As listed in Table S4, the interatomic distances Ta–P (2.59582 Å; due to symmetry identical: Li1–P1, Li2–Ta, Li1–Li2), Li2–P (2.9974 Å), and P–P distances (4.239 Å) are within the range of reported ones of related ternary compounds such as Li_14_ *Tt*P_6_ (*Tt* = Si, Ge, Sn) and ωLi_9_ *Tr*P_4_ (*Tr* = Al, Ga, In).^6,8,9^ The coordination polyhedra are depicted in Figure S10. The large atomic displacement parameters of 0.17(1) Å^2^ observed for the Li2 atoms located in the octahedral voids (Wyckoff position 4b) are commonly encountered in lithium-rich phosphidotrielates and -tetrelates.^6,8,9^ This behavior can be attributed to the off-center position of the lithium atom within the octahedral void, where it is displaced toward a triangular face of the octahedron rather than occupying the geometric center.^4,5^ In contrast, the lithium atoms in Li_7_TaP_4_, which occupy only tetrahedral voids, exhibit significantly lower atomic displacement parameters of 0.011(2) Å^2^, reflecting a more constrained environment. In the case of Li_9.5_Ta_0.5_P_4_, a split-site model for the Li2 position could not be realized, and the observed large displacement parameter may therefore reflect a static or dynamic positional disorder.

DSC measurement of the reactive mixture obtained after the ball milling procedure with composition “Li_7_TaP_4_” shows several signals that cannot be assigned to individual transitions. The powder obtained after DSC shows the formation of the phase-pure ternary phase (Figures S4 and S11). Phase-pure Li_7_TaP_4_ undergoes an endothermic decomposition at 717 °C (Figure S12) forming the lithium-rich phase Li_9.5_Ta_0.5_P_4_, the binary compound TaP, and products that could not be identified (Figure S5). The reactive mixture with the composition “Li_9.5_Ta_0.5_P_4_” shows an endothermic signal at 550 °C, which indicates the formation of the lithium-poor phase Li_7_TaP_4_ (Figure S13). At 636 °C, Li_9.5_Ta_0.5_P_4_ is formed during an exothermic process. Phase analysis via powder XRD shows the incomplete formation of Li_9.5_Ta_0.5_P_4_ with Li_3_P and elemental Ta as impurities (Figure S6). Phase-pure Li_9.5_Ta_0.5_P_4_ does not show any thermal signal up to 1000 °C (Figure S14), which can also be seen by its remaining purity in the powder XRD after the DSC analysis (Figure S7).

The investigation of Li_12–5*x*_Ta_*x*_P_4_ for *x* = 0.25 and 0.75 indicated a very narrow phase width for Li_9.5_Ta_0.5_P_4_ but due to uncertainties in the weighing procedure affecting the composition parameter *x*, no further investigations were carried out (for details, see Experimental Section).

### Raman Spectroscopy

The Raman spectra of powdered Li_7_TaP_4_ and Li_9.5_Ta_0.5_P_4_ recorded at ambient temperature are depicted in Figure 3a,b, respectively. Additionally, a theoretical Raman spectrum was calculated for Li_7_TaP_4_. The Raman spectrum of Li_7_TaP_4_ closely matches the calculated spectrum. The most prominent peaks correspond to various stretching and bending modes of the TaP_4_ tetrahedra and are summarized in Table S5. The signals at 174, 205, and 407 cm^–1^ correspond to symmetric and asymmetric bending and symmetric stretching, respectively. These modes are invariably accompanied by lattice vibrations of Li. The signal at 131 cm^–1^ could not be clearly assigned. The Raman spectrum of Li_9.5_Ta_0.5_P_4_ contains one distinct and two broad signals at 388 cm^–1^ and at 210 as well as 460 cm^–1^, respectively. Such a broadening arises from the disorder in the structure and reflects the fact that locally different coordination polyhedra of the TaP_4_ unit occur. The most intense signal is attributed to the symmetric stretching mode and is subject to a red shift compared to Li_7_TaP_4_. This also correlates with the observed increased Ta–P bond length in Li_9.5_Ta_0.5_P_4_ as already described in the structural part above. Comparison with literature-reported compounds containing isolated TaP_4_ units reveals similar vibrational frequencies. In the Raman spectrum of Na_7_TaP_4_, the symmetric stretching mode occurs at 378 cm^–1^.^48^ The IR absorption band of the tetrahedral units in Na_5_SrTaP_4_, corresponding to asymmetric stretching or bending, is observed at 370 cm^–1^.^51^ In contrast, the vibrational modes in the newly discovered lithium tantalum phosphides exhibit a blue shift to higher wavenumbers. This shift could be attributed to the influence of the Li being more electropositive than Na and Li having a higher charge density, which leads to a stronger polarization of the P–Li bond that might result in increasing the vibrational frequency.

**Figure 3:**
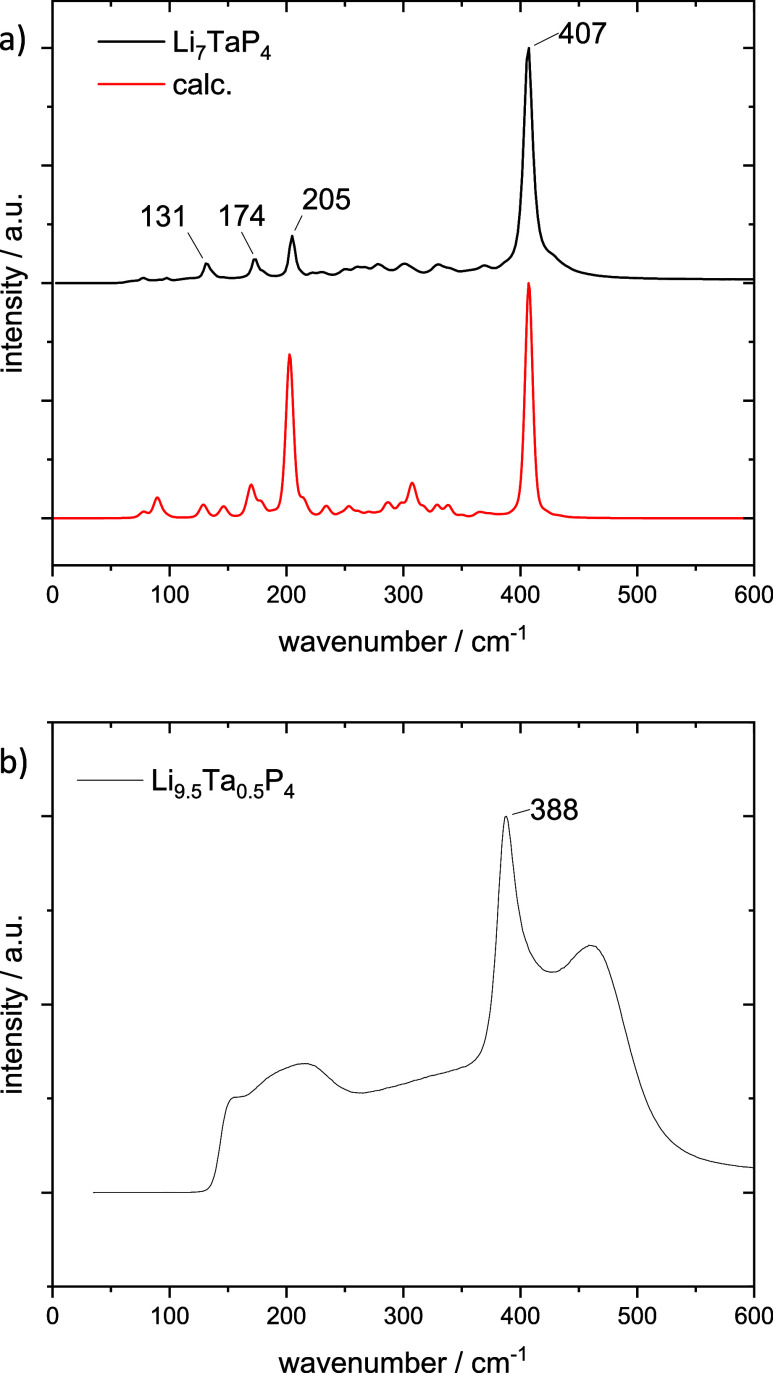
Measured Raman spectra of the product a) Li_7_TaP_4_ and b) Li_9.5_Ta_0.5_P_4_ are depicted in black. Additionally depicted is the calculated Raman spectrum of Li_7_TaP_4_ in red.

### MAS-NMR Spectroscopy

Both compounds were analyzed by ^6^Li- and ^31^P-MAS-NMR spectroscopy. The ^6^Li-MAS-NMR spectrum of Li_7_TaP_4_ reveals three adjacent signals at chemical shifts of 3.85, 3.16, and 2.23 ppm in a ratio of 3:3:1, representing the three crystallographically distinct lithium atom positions Li2 (24*d*), Li3 (24*d*), and Li1 (8*c*), respectively (Figure 4a). The least intense signal at 2.23 ppm is assigned to the lithium atom at crystallographic position 8*c*, while the other two signals can be attributed to Li2 and Li3, respectively. The two distinct signals at chemical shifts of 3.85 and 3.16 ppm arise from the differing environments of the lithium atoms located at different Wyckoff positions, an exact assignment cannot be made with the available data. The observation of well-resolved, site-specific signals suggests limited lithium-ion mobility and, consequently, low ionic conductivity.^41,52,53^ The corresponding ^31^P-MAS-NMR spectrum is depicted in Figure 4b. The two signals at 158 and 189 ppm are assigned to Li_7_TaP_4_. These signals exhibit a multitude of rotational sidebands (*) with high intensities. The quadrupolar nature of the tantalum nucleus justifies the asymmetrical arrangement of the rotational sidebands. The ratio of the integrals of the signals approximately corresponds to the expected ratio of 3:1, which matches the ratio expected from the multiplicity of the crystallographic P positions as determined from the structure refinement. The chemical shift is similar to the related compound Na_7_TaP_4_ but is significantly downfield-shifted compared to the chemical shift range of −250 to −300 ppm observed for solids comprising isolated *E*P_4_ tetrahedra (*E* = Al, Si, Ge).^3,4,9,27^ A less intense signal is observed at a chemical shift of 10.9 ppm, which can be attributed to phosphate groups that appear as a minor impurity.^54,55^ Additionally, two signals of weak intensity are visible at −338 and −91.9 ppm, which can be assigned to the chemical shift of Li_9.5_Ta_0.5_P_4_ and one of its rotational sidebands, as discussed below.

**Figure 4:**
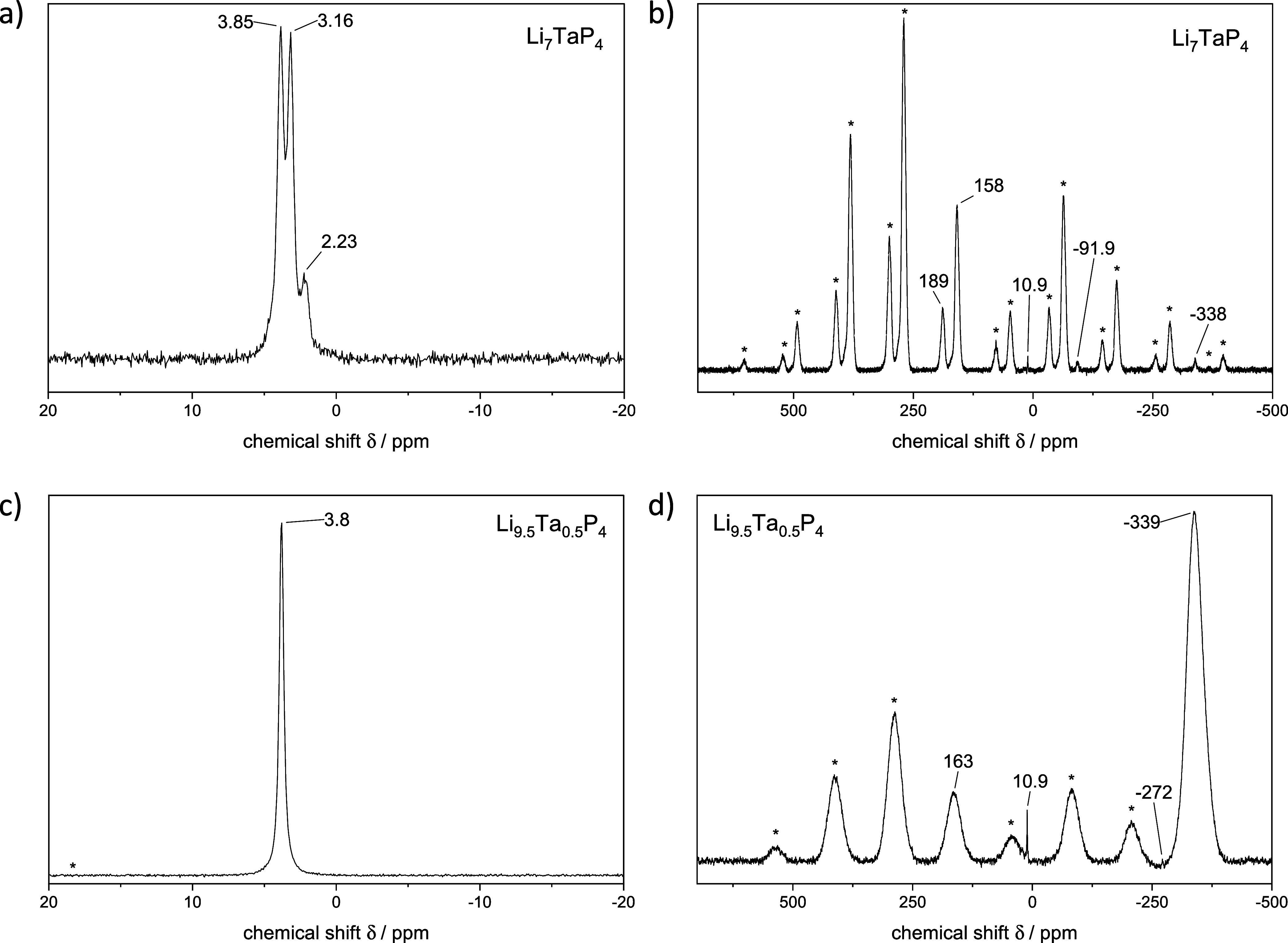
^6^Li MAS-NMR spectra of a) Li_7_TaP_4_ and c) Li_9.5_Ta_0.5_P_4_. ^31^P MAS-NMR spectra of b) Li_7_TaP_4_ and d) Li_9.5_Ta_0.5_P_4_. Rotational sidebands are marked with an *.

The ^6^Li-MAS-NMR spectrum of Li_9.5_Ta_0.5_P_4_ shows one signal with a chemical shift of 3.8 ppm with an emerging rotational sideband, which occurs asymmetrically due to the quadrupole core of tantalum (Figure 4c). Therefore, both lithium positions, Li1 and Li2, appear with the same chemical shift as is known for compounds with high lithium mobility such as Li_14_ *Tt*P_6_ (*Tt* = Si, Ge, Sn).^8,9^ The corresponding ^31^P-MAS-NMR spectrum exhibits two significantly broadened signals with high intensity at 163 and −339 ppm with rotational sidebands (Figure 4d) and two sharp signals with low intensity at 10.9 and −272 ppm. The latter result from very small amounts of phosphate and Li_3_P, respectively.^56^ The broad signals are assigned to the main product Li_9.5_Ta_0.5_P_4_ and the significant broadening of the signals is attributed to the disorder of the Li and Ta atoms. Consequently, the phosphorus atoms experience various environments due to the statistical Li/Ta occupation of tetrahedral sites and the partial occupancy of octahedral sites with Li atoms. Despite the crystallographically identical phosphorus atoms within the structure, two distinct phosphorus signals are observed, which indicate that locally some ordering occurs, which, however, cannot be resolved in the small unit cell observed by the diffraction experiment. This behavior is also observed in the related compounds Li_14_ *Tt*P_6_ (*Tt* = Si, Ge, Sn).^8,9^

### Impedance Spectroscopy and Chronoamperometry

The electrochemical properties of Li_7_TaP_4_ and Li_9.5_Ta_0.5_P_4_ were investigated by temperature-dependent impedance spectroscopy and chronoamperometry (CA) in an ion-blocking configuration at a pressure of 150 MPa. Impedance spectra were recorded at different temperatures (10, 25, 40, 55, and 70 °C) to determine the activation energies of the lithium-ion migration processes. The Nyquist plot of Li_7_TaP_4_ features one semicircle in the high-frequency regime and a second flattened semicircle in the low-frequency region (Figure 4a). The absence of a clear polarization suggests that this compound is a mixed ion-electron conductor. The impedance response can be described as a series of two parallel circuits of a resistor and a constant phase element (*R*/*Q*), with the first *R*/*Q* representing both intragrain and grain boundary contributions to the lithium-ion transport and the second *R*/*Q* corresponding to the electronic conductivity. Intragrain and grain processes could not be further resolved, and thus, only the total ionic resistance of the sample could be determined. The resulting *Q* value is 3 × 10^10^ F s^α–1^. The fitted α values are in the range of 0.66–0.7 for three independent measurements of three different samples. The ionic conductivity was determined to be σ_Li_ = 1.4(5) × 10^7^ S cm^–1^ at 25 °C. The activation energy for lithium-ion transport was determined by temperature-dependent impedance spectroscopy between 10 and 70 °C revealing an *E*_A_ of 0.48(1) eV. DC polarization measurements with steps at 50, 100, and 150 mV yield an electronic conductivity of 1.9(7) × 10^7^ S cm^–1^ at 25 °C.

The impedance spectra of Li_9.5_Ta_0.5_P_4_ are displayed in Figure 5b and feature a semicircle at high frequencies and a low-frequency tail. This behavior can be described by a series of parallel circuits of a resistor and a constant phase element and another constant phase element. *R*/*Q* corresponds to the ion transport including intragrain and grain boundary contributions, which could not be separated. The *Q* connected in series describes the electrode polarization. The fits reveal *Q* and α values of 2.2 × 10^9^ F s^α–1^ and 0.69 at 25 °C, respectively. The measured ionic conductivity σ_Li_ is 3.5(4) × 10^4^ S cm^–1^ at 25 °C with an activation energy for lithium-ion transport *E*_A_ of 0.298(6) eV. The obtained electronic conductivity by DC polarization equals 3(1) × 10^7^ S cm^–1^ at 25 °C. A summary of the values for Li_7_TaP_4_ and Li_9.5_Ta_0.5_P_4_ is given in Table 3.

**Table 3:** Comparison of the Cell Parameter *a*, the Ionic and Electronic Conductivities σ_Li_ and σ_el_ and the Activation Energy *E*_A_ of Li_7_TaP_4_ and Li_9.5_Ta_0.5_P_4_ at Ambient Temperature

	Li_7_TaP_4_	Li_9.5_Ta_0.5_P_4_
*a*/Å ( $\frac{a}{2}$ /Å)	11.80503 (5.902515)^a^	5.994796
σ_Li_/S cm^–1^	1.4(5) × 10^–7^	3.5(4) × 10^–4^
σ_el_/S cm^–1^	2 × 10^–7^	3 × 10^–7^
*E*_A_/eV	0.48(1)	0.298(6)

a Normalized parameters in brackets.

**Figure 5:**
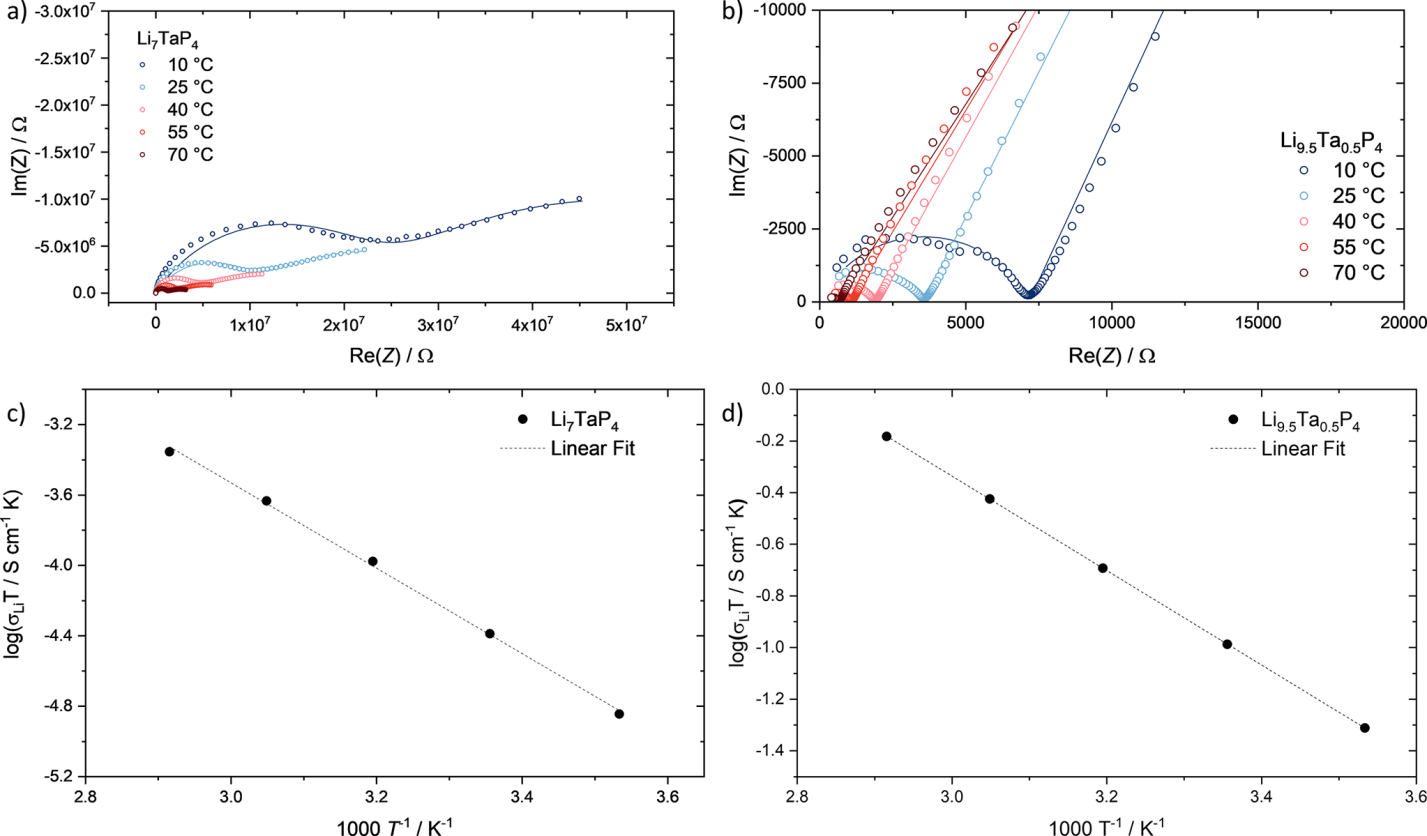
Nyquist plots in the temperature range between 10 and 70 °C of a) Li_7_TaP_4_ and b) Li_9.5_Ta_0.5_P_4_. Arrhenius plot of the product of conductivity and temperature (σ*T*) obtained during the heating cycle for c) Li_7_TaP_4_ and d) Li_9.5_Ta_0.5_P_4_. The linear fit was used to obtain the activation energy *E*_A_.

## Conclusion

The straightforward synthesis of phase-pure microcrystalline powders enables a comparison of the structures and properties of Li_7_TaP_4_ and Li_9.5_Ta_0.5_P_4_. At lower Li content, Li_7_TaP_4_ appears with fully ordered Li and Ta positions—thus fully ordered TaP_4_ tetrahedra—and no occupation of octahedral voids. The structure can be understood as an ordering variant of the Li_2_O type forming a 2 × 2 × 2 supercell. In contrast with a higher and lower amount of Li and Ta, respectively, Li_9.5_Ta_0.5_P_4_ crystallizes in a cubic cell with smaller cell parameters with Ta and Li statistically occupying the tetrahedral voids and octahedral voids are partially filled with Li, thus forming a defect variant of the Li_3_Bi type. The normalized cell parameters of the two lithium phosphidotantalates increase with a higher number of lithium atoms, which reflects the fact that the higher total number of atoms in the unit cell in Li_9.5_Ta_0.5_P_4_ dominates the cell volume.

A comparison of the void occupancies in Li_7_TaP_4_ and Li_9.5_Ta_0.5_P_4_ reveals that the tetrahedral voids are energetically preferred and are fully occupied in both compounds. In contrast, the octahedral voids remain unoccupied in Li_7_TaP_4_, whereas they exhibit ∼50% occupancy in Li_9.5_Ta_0.5_P_4_, reflecting the higher lithium content in this composition. These changes in lithium-ion density and occupation of the octahedral voids lead to an increase of the ionic conductivity by 3 orders of magnitude. Such a change has already been observed in the case of Li_5_SnP_3_ (= Li_6.67_Sn_1.33_P_4_) and Li_14_SnP_6_ (= Li_9.33_Sn_0.67_P_4_). Formal substitution of “Sn^4+^” by four Li^+^ in Li_5_SnP_3_ results in a significant enhancement of ionic conductivity, increasing from 3.2 × 10^7^ S cm^–1^ for Li_5_SnP_3_ to 9.3 × 10^4^ S cm^–1^ for Li_14_SnP_6_.^10^ In analogy to Li_7_TaP_4_ and Li_9.5_Ta_0.5_P_4_ presented here, the transition from Li_6.67_Sn_1.33_P_4_ to Li_9.33_Sn_0.67_P_4_ leads to an increase of the octahedral void occupation from 0% to 50%.

In summary, the two new ternary lithium phosphidotantalates, Li_7_TaP_4_ and Li_9.5_Ta_0.5_P_4_ extend the series of ternary lithium phosphides. Their difference in void occupancy and lithium-ion density leads to ionic conductivities that differ by 3 orders of magnitude.

## Supplementary Material



## Notes

Additional crystallographic details, XRD patterns, assignment of the Raman signals, DSC diagrams, DC polarization curves and calculated band structure (PDF)
