# Supplementary material for: Boosting the Lithium-Ion Conductivity in Li7TaP4 by Aliovalent Li versus Ta Substitution by Three Orders of Magnitude
Source: Inorg Chem. 2025 Aug 11;64(33):16902–11. doi: 10.1021/acs.inorgchem.5c02167 (PMC12381850; doi:10.1021/acs.inorgchem.5c02167)
Supplement: Supplementary file 1 [file ic5c02167_si_001.pdf]

Supporting Information

Boosting the Lithium-Ion Conductivity in Li_7TaP_4 by Aliovalent Li versus Ta Substitution by three Orders of Magnitude

Samuel Merk[a,b], Simon Kollmannsberger[a], Sabine Zeitz[a], Volodymyr Baran[c], Anatoliy Senyshyn[d], Thomas F. Fässler*[a]

[a] Technical University of Munich (TUM), TUM School of Natural Sciences, Department of Chemistry, Chair of Inorganic Chemistry with Focus on New Materials, Lichtenbergstraße 4, D-85748 Garching, Germany

[b] TUMint.Energy Research GmbH, Lichtenbergstraße 4, D-85748 Garching, Germany

[c]Deutsches Elektronen Synchrotron (DESY), Notkestr. 85, 22607 Hamburg, Germany

[d]Research Neutron Source Heinz Meier-Leibnitz (FRM II), Technische Universität München, Lichtenbergstraße 4, 85747 Garching bei München, Germany

*Corresponding author: thomas.faessler@lrz.tum.de

Contents

Details of the crystal structure determination of Li_7TaP_4 and $Li_{9.5}Ta_{0.5}P_4$ 2

Powder X-ray diffraction 5

Raman Spectroscopy 9

Thermal analysis 11

Electrochemical Analysis 13

Band structure 14

Details of the crystal structure determination of Li_7TaP_4 and $Li_{9.5}Ta_{0.5}P_4$

Results of the Rietveld refinement of Li_7TaP_4 via synchrotron powder diffraction at 293 K

Table S1. Atomic coordinates and isotropic displacement parameters of Li_7TaP_4.

Atom	Wyck. position	x	y	z	s.o.f.	U / Å^2
Li1	8*c*	0.367(1)	0.367(1)	0.367(1)	1	0.011(2)
Li2	24*d*	0.382(2)	0.140(2)	0.116(2)	1	0.011(2)
Li3	24*d*	0.368(2)	0.3709(20)	0.1185(17)	1	0.011(2)
Ta	8*c*	0.12809(6)	0.12809(6)	0.12809(6)	1	0.0057(1)
P1	8*c*	0.2451(5)	0.2451(5)	0.2451(5)	1	0.013(2)
P2	24*d*	0.0090(4)	0.24529(18)	0.01447(18)	1	0.0039(6)

Table S2. Selected interatomic distances in Li_7TaP_4.

atom pair		count	d / Å
Li1	P1	1x	2.48(2)
	P2	3x	2.62(2)
	Li3	3x	2.94(2)
	Li2	3x	2.93(2)
Li2	P2	1x	2.52(2)
	P2	1x	2.53(2)
	P1	1x	2.54(2)
	Li3	1x	2.71(3)
	P2	1x	2.76(2)
	Li1	1x	2.94(2)
	Li3	1x	3.01(3)
	Ta1	1x	3.00(2)
	Li2	2x	3.07(3)
	Li3	1x	3.88(3)
Li3	P2	1x	2.53(2)
	P1	1x	2.56(2)
	P2	1x	2.61(2)
	P2	1x	2.65(2)
	Li2	1x	2.71(3)
	Li1	1x	2.93(2)
	Li3	2x	2.94(3)
	Li2	1x	3.01(3)
	Ta1	1x	3.05(2)
	Li2	1x	3.88(3)
Ta1	P2	3x	2.386(3)
	P1	1x	2.388(5)
	Li2	3x	3.00(2)
	Li3	3x	3.05(2)
P1	Ta1	1x	2.388(5)
	Li1	1x	2.48(2)
	Li2	3x	2.54(2)
	Li3	3x	2.56(2)
	P2	3x	3.894(6)
P2	Ta1	1x	2.386(3)
	Li3	1x	2.53(2)
	Li2	1x	2.52(2)
	Li2	1x	2.53(2)
	Li1	1x	2.62(2)
	Li3	1x	2.61(2)
	Li3	1x	2.65(2)
	Li2	1x	2.76(2)
	P1	1x	3.894(6)
	P2	2x	3.899(3)

Table S3. Atomic coordinates and isotropic displacement parameters of $Li_{9.5}Ta_{0.5}P_4$.

Atom	Wyck. position	x	y	z	s.o.f.	U / Å^2
Li1	8*c*	0.25	0.25	0.25	0.9436(7)	0.0092(3)
Ta	8*c*	0.25	0.25	0.25	0.0564(7)	0.0092(3)
Li2	4*b*	0.5	0.5	0.5	0.56(2)	0.17(1)
P	4*a*	0	0	0	1	0.0196(3)

Table S4. Selected interatomic distances in $Li_{9.5}Ta_{0.5}P_4$.

atom pair		count	d / Å
Li1|Ta	Li2	4x	2.5958(1)
	P	4x	2.5958(1)
	Li1|Ta	6x	2.9974(1)
	Li1|Ta	12x	4.2390(1)
Li2	Li1|Ta	8x	2.5958(1)
	P	6x	2.9974(1)
	Li2	12x	4.2390(1)
P	Li1|Ta	8x	2.5958(1)
	Li2	6x	2.9974(1)
	P	12x	4.2390(1)

Powder X-ray diffraction

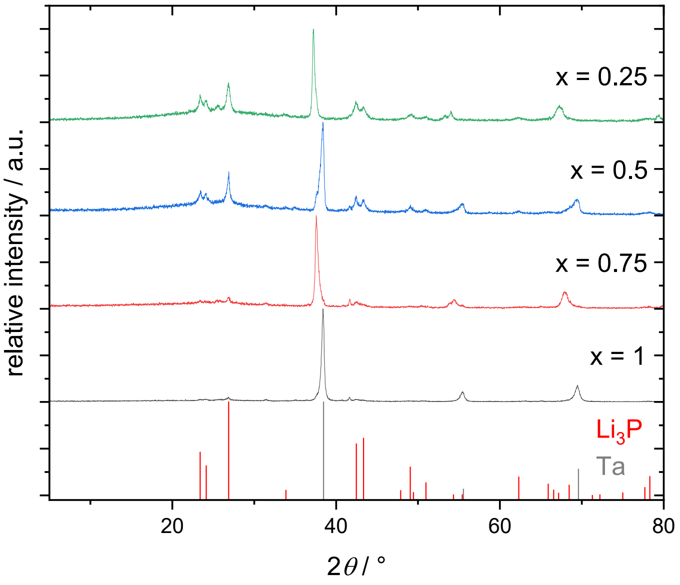

Figure S1: X-ray powder diffractograms of the reactive mixtures '$Li_{12-x}Ta_xP_4$' for x = 0.25-1. Theoretical diffractograms of Li_3P and Ta are depicted in red and grey, respectively.

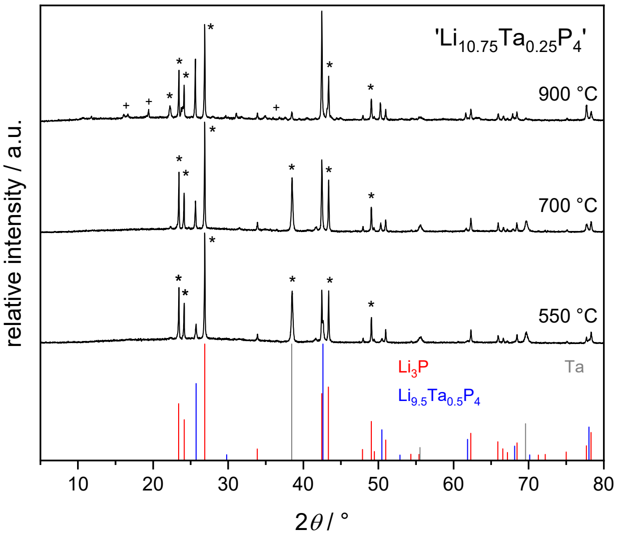

Figure S2: X-ray powder diffractograms of the reactive mixture '$Li_{10.75}Ta_{0.25}P_4$' after annealing at 550, 700 and 900 °C. Theoretical diffractograms of Li_3P, $Li_{9.5}Ta_{0.5}P_4$ and Ta are depicted in red, blue and grey, respectively. The main reflections of side phases and unidentified reflections are marked with (*) and ($^+$).

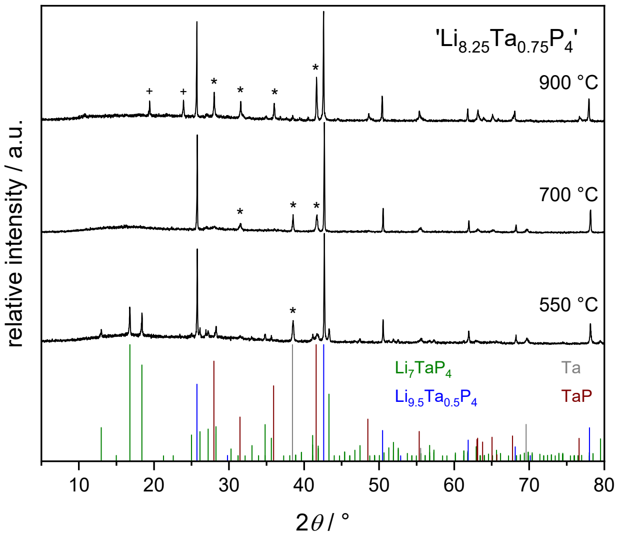

Figure S3: X-ray powder diffractograms of the reactive mixture '$Li_{8.25}Ta_{0.75}P_4$' after annealing at 550, 700 and 900 °C. Theoretical diffractograms of Li_7TaP_4, $Li_{9.5}Ta_{0.5}P_4$, Ta and TaP are depicted in green, blue, grey and brown, respectively. The main reflections of side phases and unidentified reflections are marked with (*) and ($^+$).

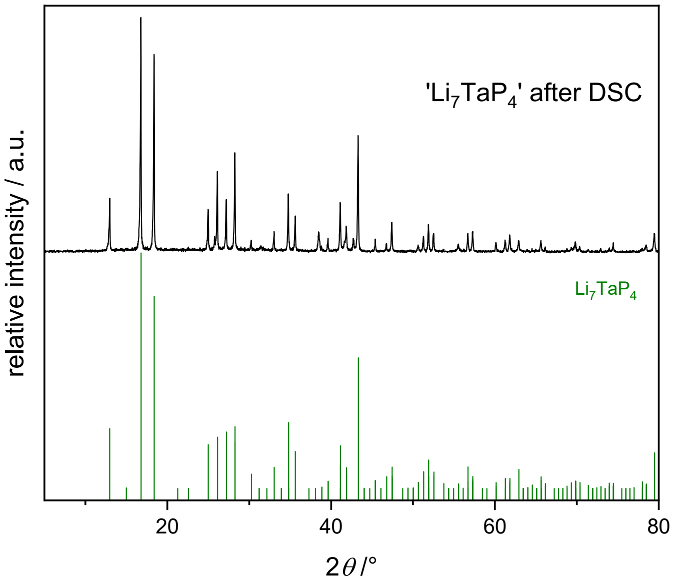

Figure S4: : X-ray powder diffractogram of the reactive mixture 'Li_7TaP_4' after DSC measurements. The sample was heated to 873 K and cooled to 423 K twice at a rate of 5 K min^{-1}. Theoretical diffractogram of Li_7TaP_4 is depicted in green.

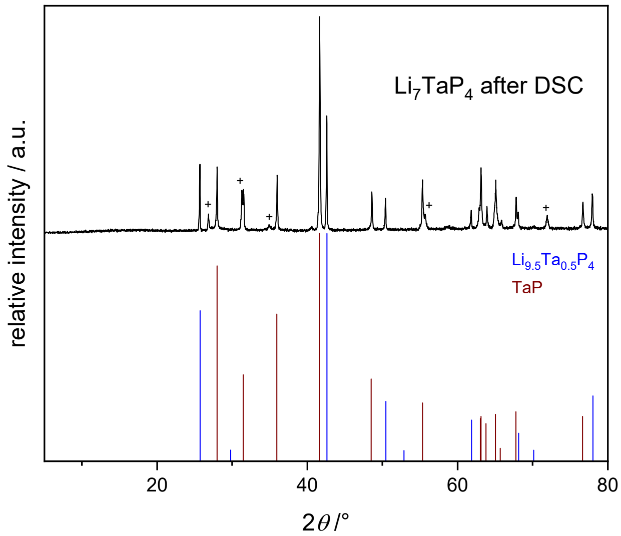

Figure S5: X-ray powder diffractogram of Li_7TaP_4 after DSC measurements. The sample was heated to 1273 K and cooled to 423 K twice at a rate of 5 K min^{-1}. Theoretical diffractograms of $Li_{9.5}Ta_{0.5}P_4$ and TaP are depicted in blue and brown, respectively. Unidentified reflections are marked ($^+$).

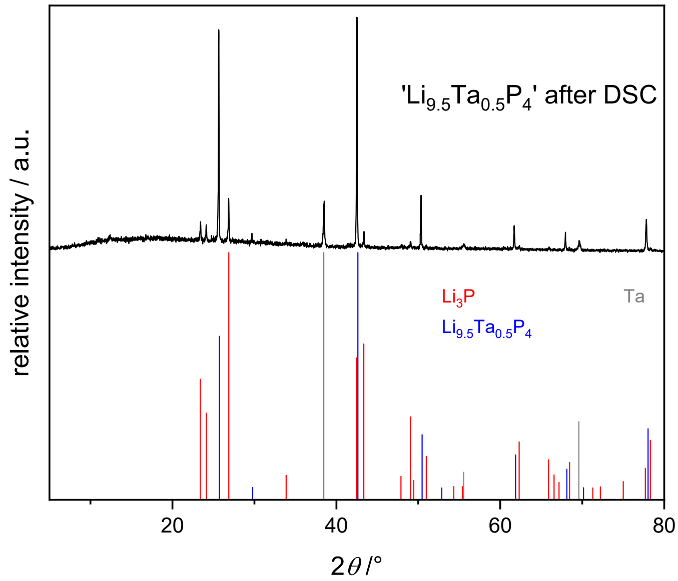

Figure S6: X-ray powder diffractogram of the reactive mixture '$Li_{9.5}Ta_{0.5}P_4$' after DSC measurements. The sample was heated to 1073 K and cooled to 423 K twice at a rate of 5 K min^{-1}. Theoretical diffractograms of $Li_{9.5}Ta_{0.5}P_4$, Ta and Li_3P are depicted in blue, grey and red, respectively.

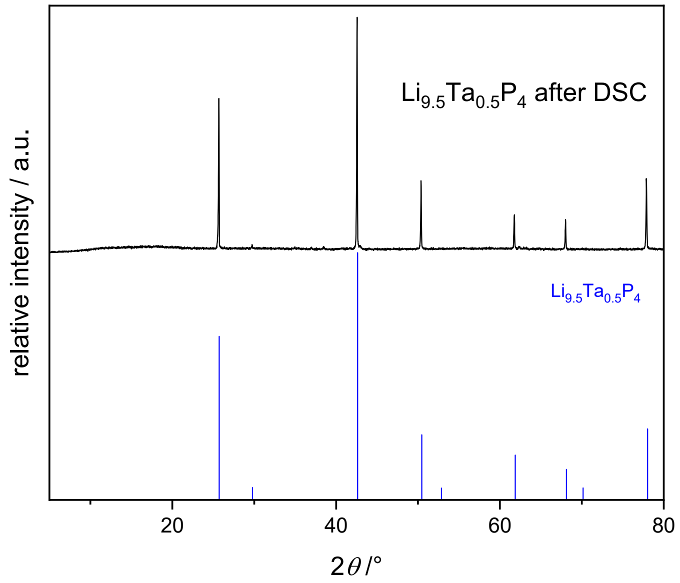

Figure S7: X-ray powder diffractogram of $Li_{9.5}Ta_{0.5}P_4$ after DSC measurements. The sample was heated to 1273 K and cooled to 423 K twice at a rate of 5 K min^{-1}. Theoretical diffractograms of $Li_{9.5}Ta_{0.5}P_4$ is depicted in blue.

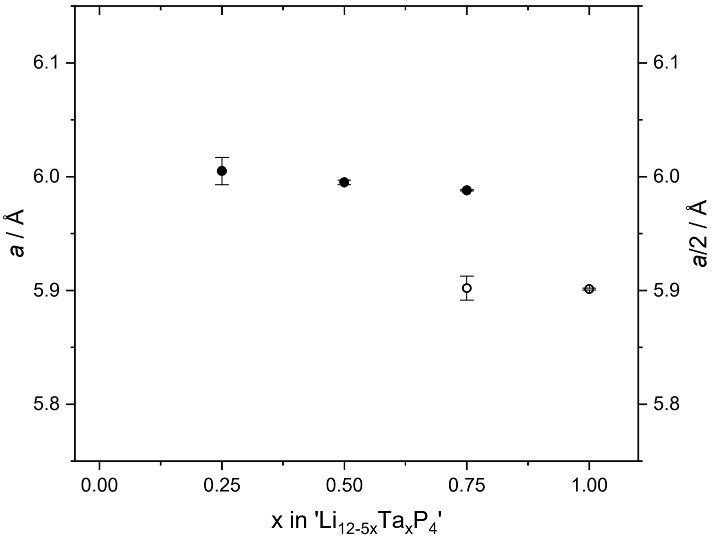

Figure S8: By indexing obtained normalized and conventional lattice parameters of Li_7TaP_4 (empty circles) and $Li_{9.5}Ta_{0.5}P_4$ (filled circles), respectively, from annealing of the reactive mixtures '$Li_{10.75}Ta_{0.25}P_4$' (x = 0.25) and '$Li_{8.25}Ta_{0.75}P_4$' (x = 0.75). The error bars represent the range defined by the 3σ-rule. For x = 0.75, Li_7TaP_4 and $Li_{9.5}Ta_{0.5}P_4$ were present as separate phases in the sample.

Raman Spectroscopy

Table S5: The most prominent peaks of the Raman spectrum of Li_7TaP_4. The column “comment” verbalizes the visual impression obtained by Jmol.

wavenumber / cm^{-1}	type(s) of modes	comment
174	bending	symmetric, scissoring
205	bending	asymmetric, twisting
407	stretching	symmetric

Coordination polyhedra

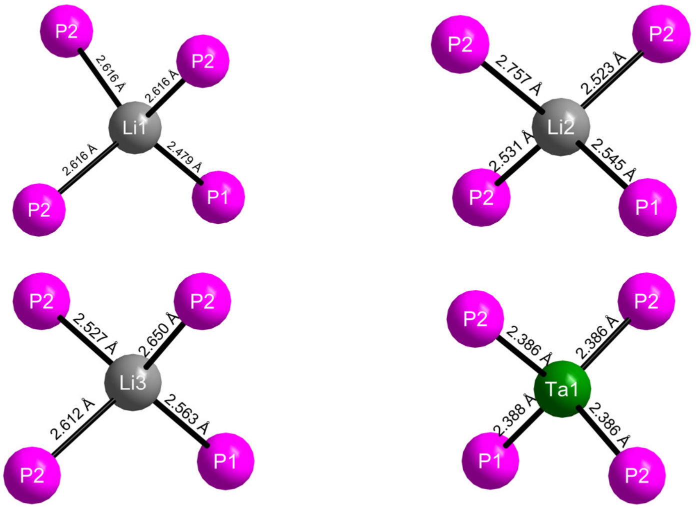

Figure S9: Coordination polyhedra of the different atomic positions Li1, Li2, Li3 and Ta1 of Li_7TaP_4.

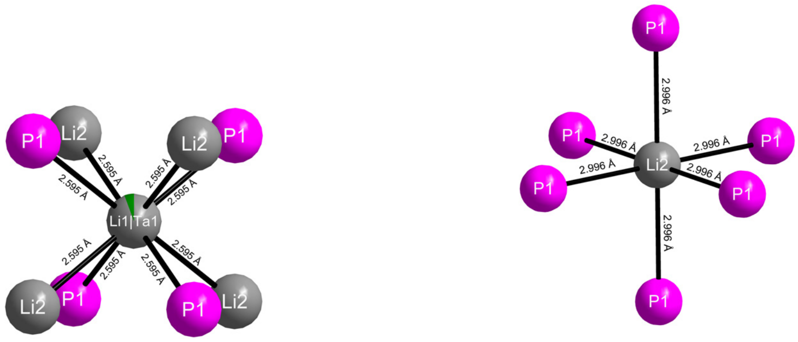

Figure S10: Coordination polyhedra of the different atomic positions Li1/Ta and Li2 in $Li_{9.5}Ta_{0.5}P_4$.

Thermal analysis

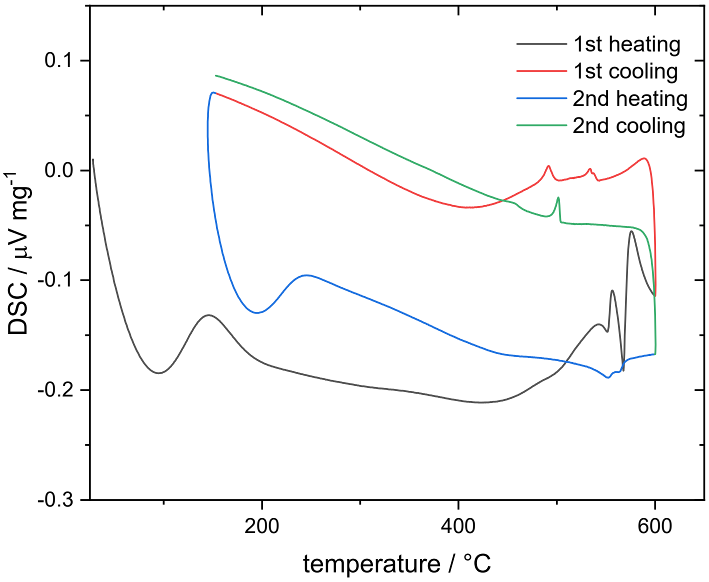

Figure S11: DSC measurement of the reactive mixture ‘Li_7TaP_4’.

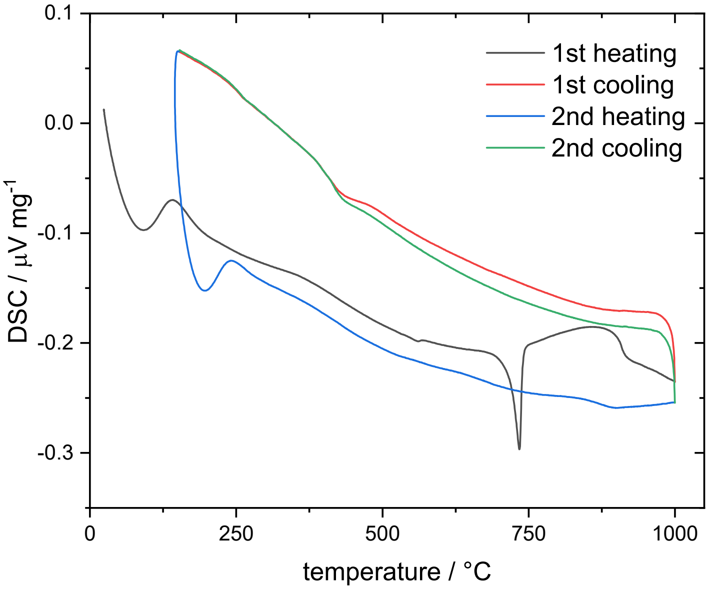

Figure S12: DSC measurement of Li_7TaP_4.

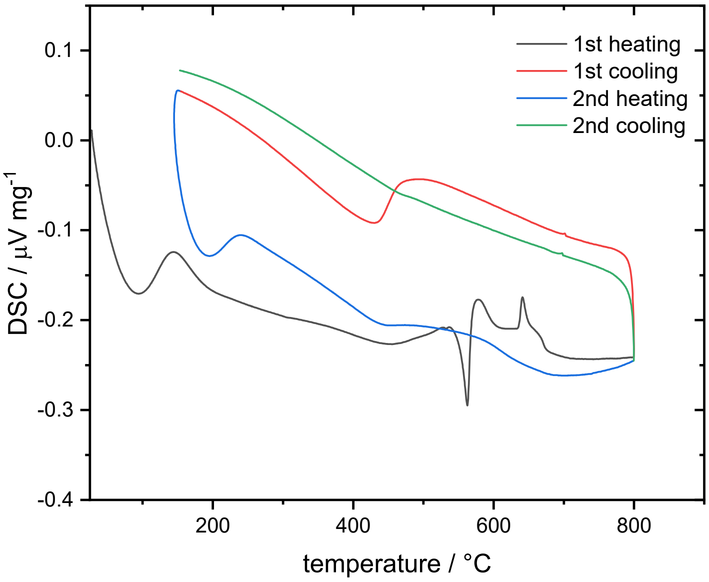

Figure S13: DSC measurement of the reactive mixture ‘$Li_{9.5}Ta_{0.5}P_4$’.

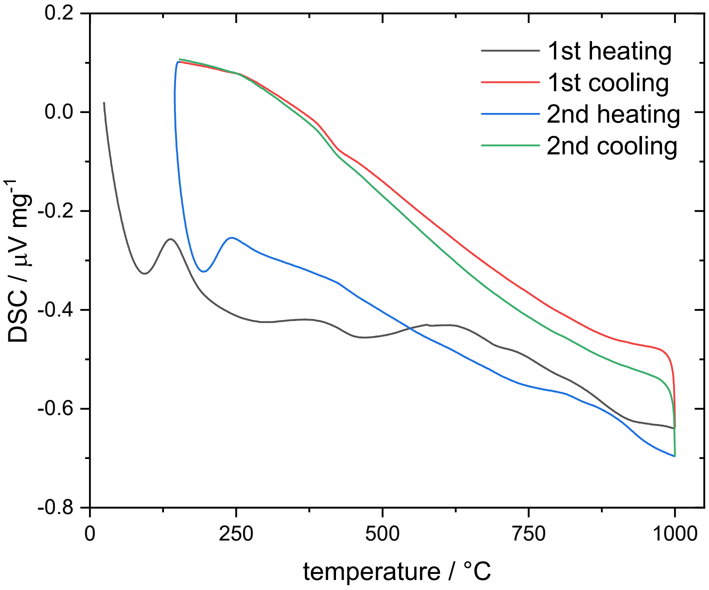

Figure S14: DSC measurement of $Li_{9.5}Ta_{0.5}P_4$.

Electrochemical Analysis

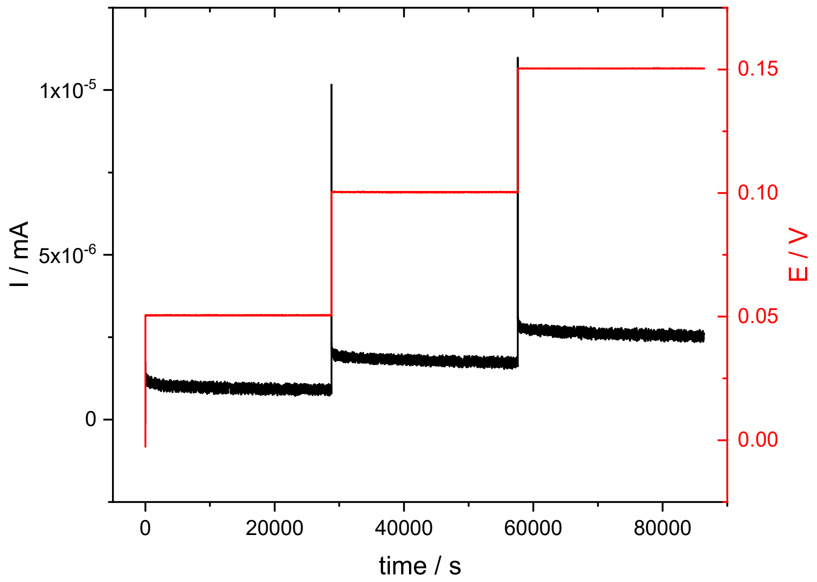

Figure S15: DC polarization of Li_7TaP_4.

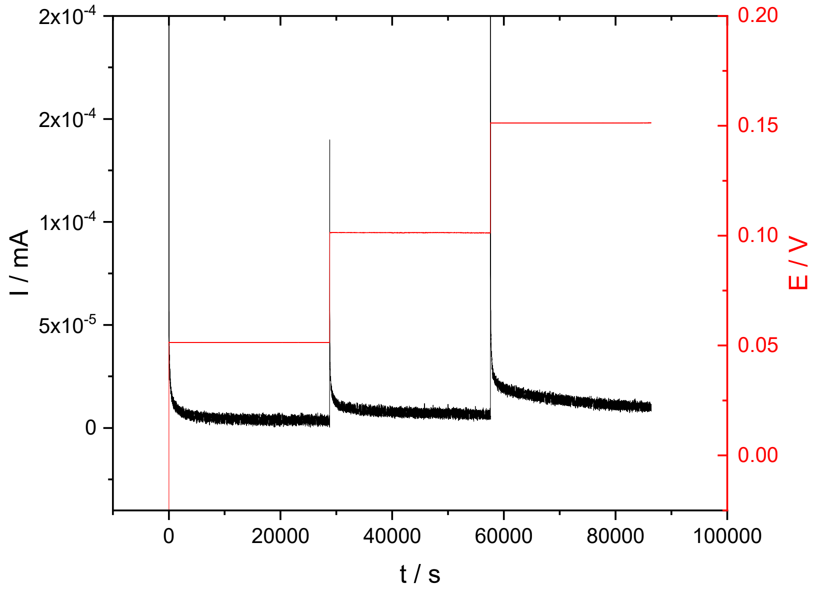

Figure S16: DC polarization of $Li_{9.5}Ta_{0.5}P_4$.

Band structure

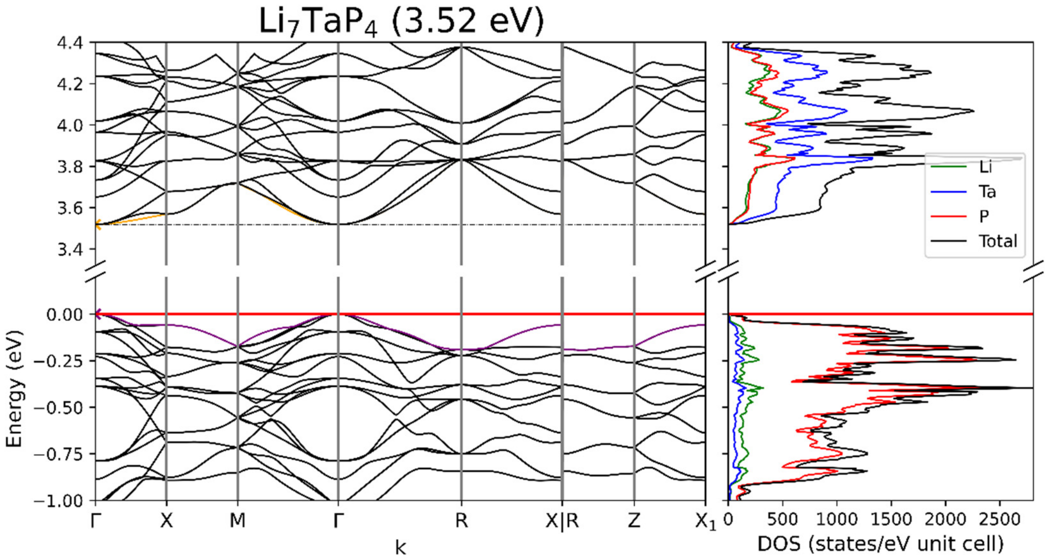

Figure S17: Band structure and atom projected DOS of Li_7TaP_4 with a direct band gap of 3.52 eV.
